# Evaluation of the Analgesia Nociception Index (ANI) as an indicator of discomfort in deeply sedated, prepubescent patients

**DOI:** 10.1007/s10877-025-01355-2

**Published:** 2025-09-09

**Authors:** Alexis Barat, Anne Wojtanowski, Hélène Behal, Mathilde Flocteil, Stéphane Leteurtre, Julien De Jonckheere, Morgan Recher

**Affiliations:** 1 Pediatric Intensive Care Unit, CHU Lille, 59000 Lille, France; 2 Univ. Lille, CHU Lille, ULR 2694 - METRICS: Évaluation Des Technologies de Santé Et Des Pratiques Médicales, 59000 Lille, France; 3 CHU Lille, CIC-IT 1403, 59000 Lille, France; 4 Biostatistics Department, CHU Lille, 59000 Lille, France

**Keywords:** Parasympathetic evaluation, Analgesia Nociception Index, Analgesia, Pain, Pediatric critical care

## Abstract

The Analgesia Nociception Index (ANI) has been used to assess discomfort in anesthetized adults. The COMFORT Behavior Scale (CBS) is recommended for assessing discomfort in intubated and sedated children. The primary objective of the present study was to assess the validity and performance of the ANI as an indicator of discomfort in intubated, ventilated children in a pediatric intensive care unit (PICU). A prospective, non-interventional, single-center pilot study was conducted between June 1st, 2021, and November 31st, 2023. Intubated, sedated, prepubescent patients aged between 2 and 10 years (for girls) or between 2 and 12 years (for boys) were included. The instantaneous ANI (ANIi) and the mean ANI (ANIm) were recorded continuously during care procedures. Data were analyzed before (period (P)1), during (P2) and after (P3) care procedures. 50 patients were included; the median (interquartile range [IQR]) age was 7 [4; 9] years. The ANIi decreased significantly between P1 and P2 (median [IQR]: 59 [45; 80] vs. 33 [26; 42], respectively; p < 0.0001) and increased significantly between P2 and P3 (33 [26; 42] vs. 51 [33; 72], respectively; p < 0.0001). The CBS score increased significantly between P2 and P3 (median [IQR]: 10 [7; 13] vs. 12 [8; 15], respectively; p < 0.0001). The ANIi was able to discriminate between over-analgosedation (defined as a CBS score < 10) and normal analgosedation (AUROC = 0.694 during P1).The ANI might be a good candidate for assessing discomfort in intubated, prepubescent patients in the PICU.

*Trial Registration*: NCT04913038

## Introduction

In intensive care units (ICUs), pain management remains a major challenge. The literature data show that a significant proportion of pediatric inpatients (particularly those on conventional wards) experience painful episodes; this observation highlights the importance of recognizing and adequately treating pain [1]. However, the assessment of discomfort in sedated, intubated patients in pediatric ICUs (PICUs) is complicated by the limitations of the currently available, non-continuous assessment scales (such as the COMFORT Behavior Scale (CBS)), which are largely dependent on the evaluator's level of experience [2]. Moreover, effective pain relief sometimes comes at the cost of excessive analgosedation; the latter is associated with adverse effects, including an elevated incidence of delirium and a heightened risk of withdrawal syndrome [3].

The Analgesia Nociception Index (ANI) system (MDoloris Medical Systems SAS, Loos, France) is based on the noninvasive analysis of high-frequency heart rate variability (HRV), which is mediated by the balance between the sympathetic and parasympathetic autonomic nervous systems and particularly by the respiratory sinus arrhythmia [4]. The ANI has proved its effectiveness as a marker of the nociception/antinociception imbalance in adults during surgery under general anesthesia. In this clinical setting, the ANI performed better than the hemodynamic variables (heart rate (HR) and blood pressure (BP)) usually used to assess nociception [5]. The ANI technology has also been validated in patients between 2 and 12 years during surgery under general anesthesia or in post-anesthesia care units [6–9]. Although several researchers have studied the ANI’s ability to evaluate pain in adults in the ICU [10–12], we are not aware of published reports on the use of the ANI to gauge discomfort in sedated, intubated, prepubescent patients.

Hence, the primary objective of the present study was to assess the utility of the ANI for the continuous evaluation of comfort in intubated/ventilated prepubescent patients in a PICU. The secondary objective was to determine the ANI’s threshold for detecting discomfort.

## Materials and methods

### Ethics approval

The study protocol was approved by an institutional review board (*CPP Sud Ouest et Outre Mer III*, Bordeaux, France; reference 2021-A00487-34) on April 28th, 2021. The study was registered at ClinicalTrials.gov (NCT04913038) before the start of the recruitment phase.

### Study design

The design of this prospective, observational study was similar to that of an earlier assessment of the Newborn Infant Parasympathetic Evaluation (NIPE), a variant of the ANI adapted to serve as a guide to the comfort of sedated, intubated children under the age of 3 years [13]. The present study was conducted in the PICU at Lille University Medical Center (Lille, France). Patients were recruited between June 2021 and November 2023. The inclusion criteria for patients in the PICU were age between 2 and 10 years (for girls) or between 2 and 12 years (for boys), sedation, intubation, and ventilation. This difference in puberty age threshold between boys and girls was based on established literature [14]. The main exclusion criteria were a nonsinus cardiac rhythm, treatment with atropine, the presence of a pacemaker, refusal to participate by the child’s legal representative, and conditions that contraindicated the use of the ANI (apnea, a respiratory rate ≤ 9/min, atropine injection during the procedure), the withdrawal of life support, and a poor-quality electrocardiogram.

### Conduct of the study

After the investigator had confirmed that the child’s legal representative did not object to study participation, the patients were observed during the following care procedures: washing, tracheal, pharyngeal, nasal or oral suctioning, dressing changes, catheter placement, bladder catheterization, bedsheet changes, changes in posture, venous and capillary blood gas measurements, and capillary blood glucose measurements.

Investigators and nurses were present during the care procedures. The ANI recording was initiated 10 min before the start of the care period and finished 10 min after the end of the care period. A nurse rated the CBS for each patient 5 min before the start of the care period (P1), during the care period (P2), and 5 min after the end of the care period (P3). The nurses who rated the CBS were blinded to the ANI during the recording. All nurses involved in the study have been trained to the CBS which is used routinely in the Lille university hospital PICU to evaluate patient comfort.

### Data collection

ANI recordings were extracted and analyzed by an independent research engineer. The ANI monitor displays the mean ANI (ANIm), the ANI averaged over a 3-min window) and the instantaneous ANI (ANIi, the ANI averaged over a 1-min window). The ANI varies between 0 (the highest possible level of discomfort) and 100 (the absence of discomfort). The lowest values of ANIm and ANIi during each of P1, P2 and P3 were recorded.

The CBS comprises six items (alertness, calmness-agitation, respiratory response, physical movement, muscle tone, and facial tension), each of which is rated between 0 (least intense) and 5 (most intense). A CBS score of between 6 and 10 corresponds to over-analgosedation, a score of between 11 and 17 corresponds to a comfortable, sedated state, a score between 17 and 22 corresponds to a boundary state with possible discomfort, and a score of between 23 and 30 corresponds to discomfort [15, 16].

Data on hemodynamic and respiratory variables (the maximum HR, the maximum systolic BP (SBP), the maximum diastolic BP (DBP), the mean BP, the minimum oxygen saturation, the maximum inspired oxygen fraction, the maximum tidal volume (Vt), and the maximum positive end expiratory pressure) were collected for P1, P2 and P3. We also recorded information on age, weight, the reason for admission to the PICU, the Paediatric Index of Mortality 3, the updated Pediatric Logistic Organ Dysfunction score, the dose levels of sedatives and analgesics, the number of boli of sedatives and analgesics administered, the length of the care period, and the type of care.

### Outcome measures

The study’s primary outcome was the difference between the ANI scores in P1 and the ANI scores in P2. The secondary outcomes were the difference between the ANI scores in P2 and the ANI scores in P3, the correlations between the difference in the ANI scores and the difference in the CBS score for each period, and the correlations between the minimum ANI scores and the CBS score for each period. We also studied the ANI scores’ ability to discriminate between discomfort and comfort (defined by a CBS score threshold of 17 during the care period), and between normal analgosedation and over-analgosedation (defined by a CBS threshold of 10 before the care period).

### Statistical analysis

The main objective of this exploratory study was to evaluate ANI variations between a period without care and a period with care in a prepubescent pediatric population admitted to intensive care. There were no data available in the literature allowing the sample size computation based on a statistical hypothesis. The sample size was therefore based on the recruitment capacity of the Lille university hospital PICU. Since the inclusion potential of the department was around 100 patients per year, we proposed to recruit 50 patients.

Qualitative variables were described as the number (percentage). Quantitative variables were described as the mean (standard deviation (SD)) if normally distributed, or the median [interquartile range] if not. The normality of the data’s distribution was assessed using a Shapiro–Wilk test and checked graphically on histograms. Periods were compared in a Student’s test for paired data if the data were normally distributed or in a signed rank test if not. The correlation between the difference in the ANI and the difference in the CBS score was quantified by calculation of Pearson’s coefficient (for normally distributed data) or Spearman’s coefficient (for nonnormally distributed data). A received operating characteristic (ROC) curve was used to analyze the ability to discriminate between discomfort and comfort and between over-analgosedation and normal analgosedation.

## Results

### Population

A total of 50 patients were included in the study. The median [IQR] age was 7 [4; 9], the median bodyweight was 19 (15; 28] kg, the female/male sex ratio was 0.8, and the median duration of a care procedure was 23 min [16; 36]. (Table 1). In no cases did the child’s legal representative object part in the study. No technical problems were reported. The following care procedures were documented: tracheal suctioning (n = 43), washing (n = 41), oral or nasal suctioning (n = 39), changes in posture (n = 26), massage (n = 22), bladder catheterization (n = 13), collection of a capillary blood sample (n = 13), dressing changes (n = 8), radiography or echography (n = 4), and placement of a peripheral venous catheter (n = 3).

**Table 1:** Patients’ characteristics

Age, year	7 (4–9)	
Sex (female/male)	22/28	
Weight, kg	19 (15–28)	
Reason for admission n (%)		
Medical	37 (74)	
Surgical	13 (26)	
Medical or surgical antecedents n (%)	34 (68)	
Current treatment n (%)	19 (38)	
Septic chock n (%)	2 (4)	
Head trauma n (%)	5 (10)	
PELOD-2 Day-1	5 (3;7)	
PIM III	1.57 (0.63;4.22)	
Analgesia n (%)	47 (9)	
Morphine	34 (68)	
Dose (mg/kg/day)	1 (0.52;1)	
Remifentanil	2 (4)	
Dose (µg/kg/h)	15 (13.5;16.5)	
Sufentanil	12 (24)	
Dose (µg/kg/h)	0.915 (0.475;1.275)	
Ketamine	1 (2)	
Dose (mg/kg/h)	4	
Analgesia bolus during procedure n (%)		
Morphine	5 (10)	
Dose (mg)	1 (0.7;1)	
Ketamine	1 (2)	
Dose (mg)	40	
Sufentanil	1 (2)	
Dose (mg)	5	
Paracetamol	3 (6)	
Vasopressor n (%)		
Dobutamine	2 (4)	
Dose (µg/kg/min)	7.5 (6.25–8.75)	
Norepinephrine	10 (20)	
Dose (µg/kg/min)	0.07 (0.05–0.2)	
Milrinone	2 (4)	
Dose (µg/kg/min)	0.405 (0.4025–0.4075)	
Sedation n (%)	50 (100)	
Midazolam	43 (86)	
Dose (µg/kg/min)	1 (0.5–1.5)	
Propofol	10 (20)	
Dose (mg/kg/h)	2.5 (1.25–4.875)	
Dexmedetomidine	8 (16)	
Dose (µg/kg/h)	0.6915 (0.6147–1)	
Hydroxyzine	1 (2)	
Sedation bolus during procedure n (%)		
Midazolam	12 (24)	
Dose (mg)	2 (1.7–3)	
Propofol	3 (6)	
Dose (mg)	50 (42.5–55)	

kg kilograms; mg/kg/day milligrams per kilo per day; µg/kg/h microgram per kilo per hour; µg/kg/min microgram per kilo per minute; mg milligram. PELOD-2 Pediatric Logistic Organ Dysfunction 2 score. PIM III Pediatric Index of Mortality 3

### Assessments of the ANI

The ANIi fell significantly between P1 and P2 (median [IQR]: 59 [45; 80] vs. 33 [26; 42], respectively; p < 0.0001), and a significant increase in the ANIi between P2 and P3 (33 [26; 42] vs. 51 [33; 72], respectively; p < 0.0001). The ANIm values significantly decreased between P1 and P2 (median [IQR]: 64 [56; 79] vs. 45 [37; 56], respectively; p < 0.0001), and the ANIm values significantly increased between P2 and P3 (45 [37; 56] vs. 51 [33; 72], respectively); p < 0.0001) (Table 2).

**Table 2:** Evolution of ANI, Comfort B scale, hemodynamic and ventilation values for each period

	P1	P2	P3	p-value P1 vs P2	p-value P2 vs P3
ANIi	59 (45;80)	33 (26;42)	51 (33;72)	** < 0.0001**	** < 0.0001**
ANIa	64 (56;79)	45 (37;56)	59 (43;77)	** < 0.0001**	** < 0.0001**
CBS	9 (7;12)	10 (7;13)	12 (8;15)	0.4	** < 0.0001**
HR, bpm	110 (90;124)	136 (115;145)	114 (94;133)	** < 0.0001**	** < 0.0001**
SBP, mmHg	103 (92;113)	116 (99;123)	108 (91;115)	** < 0.0001**	** < 0.0001**
DBP, mmHg	58 (51;67)	70 (60;81)	61 (52;75)	** < 0.0001**	** < 0.0001**
MBP, mmHg	73 (65;83)	84 (75;96)	77 (66;86)	** < 0.0001**	** < 0.0001**
SpO2, %	99 (96;100)	94 (91;97)	97 (93;99)	** < 0.0001**	**0.0035**
FiO2, %	30 (25;40)	60 (35;100)	30 (25;48)	** < 0.0001**	** < 0.0001**
Vt, mL	130 (104;201)	153 (106;230)	140 (100;210)	** < 0.001**	** < 0.0001**
PEEP, cm H2O	5 (5;7)	6 (5;8)	5.4 (5;8)	**0.025**	**0.025**

Significant results are given in bold

ANIi: Instantaneous Analgesia Nociception Index; ANIa: Average Analgesia Nociception Index; HR: Heart Rate, SBP: Systolic Blood Pressure; DBP: Diastolic Blood Pressure; MBP: Mean Blood Pressure

The CBS score did not change significantly between P1 and P2 (median [IQR]: 9 [7; 12] vs. 10 [7; 13], respectively; p = 0.4) but increased significantly between P2 and P3 (10 [7; 13] vs. 12 [8; 15], respectively; p < 0.0001) (Table 2). During P1 and P2, 30/50 (60%) patients and 33/50 (66%) patients had respectively a CBS score < 10. Neither the ANIi nor the ANIm was correlated with the CBS for any period (Table 3).

**Table 3:** Correlation between the ANI and the Comfort B scale

	P1		P2		P3	
	R	p-value	R	p-value	R	p-value
ANIi	− 0.15	0.3	0.04	0.8	0.15	0.3
ANIm	− 0.13	0.4	0.02	0.9	0.21	0.14

Significant results are given in bold

ANIi: Instantaneous Analgesia Nociception Index; ANIm: Mean Analgesia Nociception Index

With regard to hemodynamic variables, there was a significant increase in the HR between P1 and P2 (110 [90; 124] vs. 136 [115; 145], respectively; p < 0.0001), and a significant decrease between P2 and P3 (136 [115; 145] vs. 114 [94; 133], respectively; p < 0.0001). The SBP significantly increased between P1 and P2 (103 [92; 113] vs. 116 [99; 123], respectively; p < 0.0001), and decreased significantly between P2 and P3 (116 [99; 123] vs. 108 [91; 115], respectively; p < 0.0001) (Table 2).

Only 3 of the 50 patients demonstrated a behavioral response to pain (defined as a CBS score > 17); this prevented us from defining a threshold in the area under the ROC curve (AUROC) for the detection of discomfort. In P1, the AUROC for discriminating between over-analgosedation and normal analgosedation (defined by a CBS threshold of 10 before the care period) was 0.694 for the ANIi (sensitivity = 0.42, specificity = 0.87, threshold = 67%) and 0.690 for the ANIm (sensitivity = 0.39, specificity = 0.93, threshold = 76.5%).

## Discussion

The primary objective of the present study was to assess the utility of the ANI for the continuous assessment of comfort in intubated/ventilated prepubescent patients in a PICU. The ANIi and ANIm varied according to the presence or absence of painful stimuli. Indeed, the values decreased significantly during the care procedures and then significantly increased afterwards. In contrast, the CBS score did not change significantly during care procedures and was not correlated with the ANI. The hemodynamic variables changed significantly during the care procedures. The ANIi demonstrated good specificity (0.87) but low sensitivity (0.42) and a low AUROC (0.694) for detecting over-analgosedation.

Several researchers have investigated the use of the ANI to evaluate pain in the ICU. In 2016, Papaioannou et al. studied the ANI’s ability to detect pain in conscious burns patients during dressing changes. The researchers found that ANI was able to detect self-evaluated pain with an AUROC of 0.75 [10]. In our previous study of deeply sedated ICU patients, we found that the ANI was an effective pain evaluation tool but was not correlated with the Behavioral Pain Scale (BPS) [17]. In a retrospective study of deeply sedated patients with COVID-19, Boselli et al. observed a significant decrease in the ANI during closed tracheal suction but did not investigate the correlation between the ANI and the BPS [11]. In Jendoubi et al.’s investigation of tracheal suctioning in ventilated, sedated patients, the ANI and the BPS were weakly but significantly correlated (r^2^ = −0.469, *p* < 0.001) [18]. The ANI was able to predict a BPS score ≥ 5 with a sensitivity of 73%, a specificity of 62%, and a negative predictive value of 86% [18]. Chanques et al. confirmed these findings by assessing the correlation between the ANI and the BPS score during 969 routine care procedures in 110 non-comatose, non-communicating, lightly sedated ICU patients [12]. The researchers observed a significant increase in the BPS score and a significant decrease in the ANI during care procedures. The correlation between the ANI and the BPS was small but significant (r = − 0.30, p < 0.0001). An ANI ≥ 43 threshold was able to detect pain with a sensitivity of 61.4%, a specificity of 77.4%, and a negative predictive value of 90.4%. Chanques et al. concluded that ANI may be of benefit for excluding significant pain. In 2018, Chanques et al. confirmed that the ANI and BPS adequately detected responses to painful stimuli in conscious ICU patients [19]. Our deeply sedated pediatric study population differed from that of Chanques et al. and probably experienced less pain which could explain the lack of correlation between ANI and CBS; indeed, the proportion of patients with probable pain during care was 1.5% (3 out of 50) in our study and 76% in Chanques et al.’s study.

In 2020, we evaluated the ability of the NIPE (a variant of the ANI) to assess pain in sedated/intubated children under the age of 3 years (13). During the care procedure, we observed a significant decrease in the mean (SD) NIPE (from 64 (2) to 42 (1)) and a significant increase in the mean (SD) CBS score (from 12 (0) to 16 (1)). We also observed a small but significant correlation between the NIPE and the CBS (r = − 0.44, p < 0.0001). Moreover, a NIPE threshold of 53 was able to predict a CBS score of up to 17 with a sensitivity of 80% and a specificity of 73.5%. In the present study, the median CBS score increased from 9 [7;12] to 10 [7;13], and only 3 of the 50 patients were considered to have experienced discomfort during the care procedure. Moreover, 33 of the 50 patients were considered to be oversedated (a CBS score < 10) before the care procedure. This high sedation may partially explain the lack of a behavioral response to painful stimulation. In contrast, most patients demonstrated a significant decrease in the ANI and an increase in the HR and/or SBP during the procedure; this confirmed the autonomic nervous system’s response to painful stimulation. Our results highlight the limitations of third-party assessments. In this type of situation, the ANI or variations in hemodynamics might provide a surrogate marker of discomfort. These results raise questions about the validity of CBS assessments in patients under deep sedation. The literature on pain and discomfort behavioral assessment in PICU remains limited. Available studies mainly address the validation of tools such as the COMFORT scale, the Comfort Behavior Scale (CBS), the FLACC scale, and the Multidimensional Assessment Pain Scale (MAPS) [15]. Among sedated patients, the CBS is most frequently used to evaluate comfort. Unlike the COMFORT scale, the CBS excludes physiological items which have demonstrated limited reliability and validity as contributors to the total score [20, 21]. On the other hand, in the particular context of our deeply sedated population, the evaluation of the COMFORT scale which includes hemodynamic items should have led to greater variations during stimulation.

Our present results are in line with those published in 2024 by Voeltzel et al., following their study of the ANI’s ability to assess pain in ICU patients receiving neuromuscular blocking agents (NMBAs). The researchers compared the hemodynamic, ANI and behavioral responses (on the BPS) to tracheal suctioning in 20 patients treated with NMBAs vs. 20 patients not treated with NMBAs. As intended, the BPS score increased only in patients not treated with NMBAs, whereas the HR and ANI varied significantly in both populations. The ANI proved to be the most discriminant variable for pain detection in both populations [22]. Furthermore, the ANI demonstrated good specificity but poor sensitivity for the detection of oversedation. In our opinion, this result was not surprising because the ANI had been developed as a specific marker of the nociception/antinociception imbalance. Several studies of patients undergoing surgery under general anesthesia have already demonstrated that the ANI is sensitive and specific for nociception but not for the level of sedation [23]. In our study, ANI did not demonstrate good performance in detecting over-analgosedation detection (Se = 0.42, Sp = 0.87, AUCROC = 0.694).

Our study had several limitations. Firstly, calculation of the ANI depended on the quality of the electrocardiogram signal. The movements caused by care procedures might have interfere with this signal and/or caused signal loss for several seconds. Secondly, we did not standardize the frequencies or durations of the various care procedures and therefore studied a care period as a whole. Indeed, the study combines multiple care procedures with varying pain perception profiles (tracheal suctioning, washing, positioning, blood sampling, etc.) without standardization of technique, duration, or intensity which may raise question on the reproducibility of the study. However, this corresponds to routine practice and further emphasizes the ANI’s clinical relevance. Moreover, we wanted to keep the same study design as the previous study performed with NIPE for results comparability purposes [13]. Thirdly, we could not assess all the factors that might have influenced the ANI and other variables; these included environmental factors such as noise, which is common in the PICU. Fourthly, comfort was assessed solely using the CBS, without including additional assessment tools that might have been more appropriate for our deeply sedated population. Nevertheless, other behavioral assessment tools are largely comparable to CBS in terms of items. However, relying only on one comfort assessment tool may limit the generalizability of our findings. Lastly, the number of patients demonstrating a behavioral response to pain was very small; this prevented us from achieving one of our study objectives (i.e. the definition of an ANI threshold for pain detection).

## Conclusion

To the best of our knowledge, the present study is the first to have examined the utility of the ANI monitor for evaluating discomfort and sedation in intubated, sedated, prepubescent children over 2 years of age in a PICU. Our conclusions were limited by a high proportion of children who were oversedated (according to the CBS scores). Nevertheless, the significant variations in the ANIi and hemodynamic variables observed during care procedures highlighted the relevance of this tool for use with a patient population in which pain assessment is particularly challenging. Thus, the ANI appears to be a promising method for the more precise and (most importantly) continuous pain assessment in the PICU; the measurement bias is likely to the lower than that of hetero-assessment scales. However, the establishment of appropriate analgesia protocols using the ANI will require further studies.

## Data Availability

The data can be obtained on request from the authors.
